# Design, implementation and performance evaluation of multi-function boost converter

**DOI:** 10.1038/s41598-023-31293-5

**Published:** 2023-03-15

**Authors:** Marwa S. Osheba, Azza E. Lashine, Arafa S. Mansour

**Affiliations:** 1 Electrical Engineering Department, Faculty of Engineering, Menoufia University, Shebin El-Kom, Egypt; 2 Electrical Engineering Department, Faculty of Engineering, Beni-Suef University, Beni-Suef, Egypt

**Keywords:** Engineering, Electrical and electronic engineering

## Abstract

This paper introduces a single-phase multi-function converter (MFC) circuit that can perform the power electronics functions of a DC–DC, a DC–AC, an AC–AC, and an AC–DC boost converter. The power circuit of the proposed converter contains only four bi-directional switches, which is sufficient side by side with a switched logic-based control system to execute converter functions. The proposed converter can implement the boosting converter function, which is a drawback of the conventional single-phase matrix converter, which is limited to performing the buck converter functions. The mathematical analysis of each converter function is presented. A state space averaging technique is employed to obtain the time-invariant state space model for a better understanding of the converter characteristics. The frequency response approach is used to demonstrate the effect of circuit parameters on converter stability using the time-invariant state-space model. Moreover, the performance analysis of the system is checked through the building of a simulation model of the proposed converter via MATLAB/Simulink and MATLAB/Simscape toolbox integration. Additionally, the actual performance of the proposed converter is verified through a laboratory prototype experimental study. The experimental results confirm those obtained by computer simulation, clearly demonstrating the successful operation of the proposed circuit as a multi-function converter with acceptable levels of total harmonic distortion (THD).

## Introduction

Power electronic devices are numerous and cover a very wide spectrum of topics, including the ever-increasing role in integrating renewable energy sources and electric storage systems into electric power systems^1–3^. With the present developments in power electronic elements, engineers can build efficient and cost-effective circuits using control theory, modern analytical tools, and design techniques to make the present smart energy revolution possible^4–6^. This helps modernize electric power systems, allows for large photovoltaic and wind energy penetration, and consequently reduces the emission of CO_2_^7,8^. Power electronic converters represent the backbone in integration of renewable energy sources and the building of micro- and smart grids. These converters are required for the DC-DC, DC-AC, AC-DC and AC-DC power conversions and various circuit topologies can be found in the literature^9–11^.

There has been a substantial development in power electronic circuits (PEC) and their control systems over the last three decades that has enabled the present vast applications in various fields^12^. Moreover, increasing the power density of such converters attracts research attention through using high switching rates. This in turn raises the interest in decreasing the switching losses by means of soft switching techniques such as; zero-voltage and zero-current switching techniques^13,14^. Furthermore, isolated converters represent a fertile field for compensating for their limitations in conversion ratios and component ratings^15^.

Depending on the topologies used and the types of loads, power electronic circuits are capable of transferring power in one direction, i.e., unidirectional power flow from the source to the load^16^. However, due to application needs, converters were designed to allow bidirectional power flow, which simplifies the circuit topology and increases their efficiencies as they use the same power electronic components^17–19^. A bidirectional DC-DC converter for use in low-power applications is proposed with a topology based on a half-bridge on the primary and a current-fed push–pull on the secondary side of a high-frequency isolation transformer^16^. A bidirectional alternating current or direct current AC/DC–DC/AC rectifier/inverter for facilitating vehicle-to-grid integration is presented in^20,21^.

Increased electrical power consumption and increased environmental concern have prompted many studies focusing on the integration of renewable energy sources into utility grids^22^. Furthermore, multiport power electronic converters are essential in building micro- and smart grids^23–25^. However, in these applications, reducing the number of power converter counts is required to improve operation reliability and mitigate their impacts on power quality. To achieve that, many efforts have been made to develop converters that can perform more than one function with the same circuit configuration using conventional, adaptive, or artificial intelligence-based control systems^26,27^. These converters have found their place in many applications, such as electric vehicle battery chargers, alternative generation systems, uninterrupted power supply systems, and hybrid energy storage systems^28,29^. The most common type of multifunction converter is the matrix converter. The single-phase topology could complete buck DC–DC, DC–AC, AC–DC, and AC–DC functions. However, matrix converters are disabled in performing the boosting function because of the absence of any passive elements^30^. This presents a challenging issue for the researchers to work on due to the importance of boosting function.

Therefore, this paper introduces a multi-function converter (MFC) able to perform boosting DC–DC, DC–AC, AC–DC, and AC–DC power converters. The circuit employs four bi-directional switches similar to those used in Matrix converters, to manage multi-function operation via a logic-based control scheme. Also, a boosting inductor is added to the supply side to achieve the boosting function. A comparison among the proposed converter and the known ones is presented in Table 1. The equivalent circuit model could be obtained via various modeling methodologies, such as continuous-time models and discrete-time models^31^. A time-invariant state-space model is developed in this work using an averaging technique, which is used to analyse converter stability using the frequency response technique. The performance of the proposed circuit is extensively tested for the various functions by computer simulation and experimentally using a DSP-based laboratory model.

**Table 1:** Comparison among the existing converters and the proposed MFC.

Converter	DC–AC	AC–DC	AC–AC	DC–DC	Boosting function
Inverter	√				√
Rectifier		√			√
AC voltage Controller			√		√
DC Chopper				√	√
Matrix	√	√	√	√	
Proposed MFC	√	√	√	√	√

The paper is organized as follows: section “Proposed circuit topology” provides the circuit configuration; the operation modes and the mathematical modelling are introduced in section “Operation principles and mathematical modelling”. Section “State-space Stability analysis” presents the stability analysis and the converter design; sections “Simulation results” and “Experimental setup” give the simulation results and the experimental results, respectively. Finally, the manuscript is concluded in section “Conclusions”.

## Proposed circuit topology

**Figure 1:**
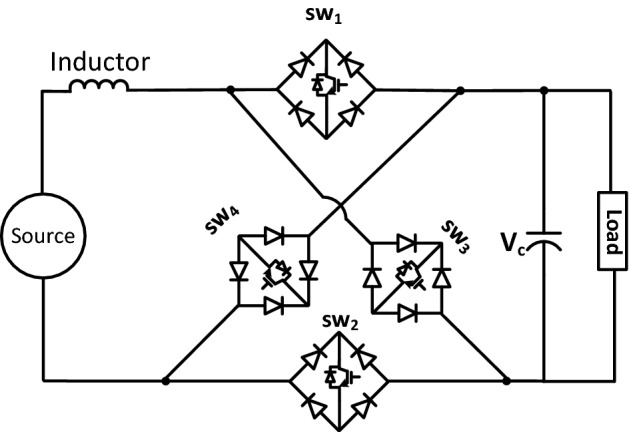
The circuit topology.

The proposed boost multi-function converter topology is shown in Fig. 1. The circuit consists of an inductor L, a capacitor C, and four bridge-based bi-directional switches; SW_1_, SW_2_, SW_3_ and SW_4_. A logic-based controller is used along with the bi-directional switches to enable the circuit operation to perform in multi-function modes. The following sections describe the operation principles, modeling, and circuit analysis used during the operation of various functions.

## Operation principles and mathematical modelling

### Circuit operation as a DC–DC boost converter

**Figure 2:**
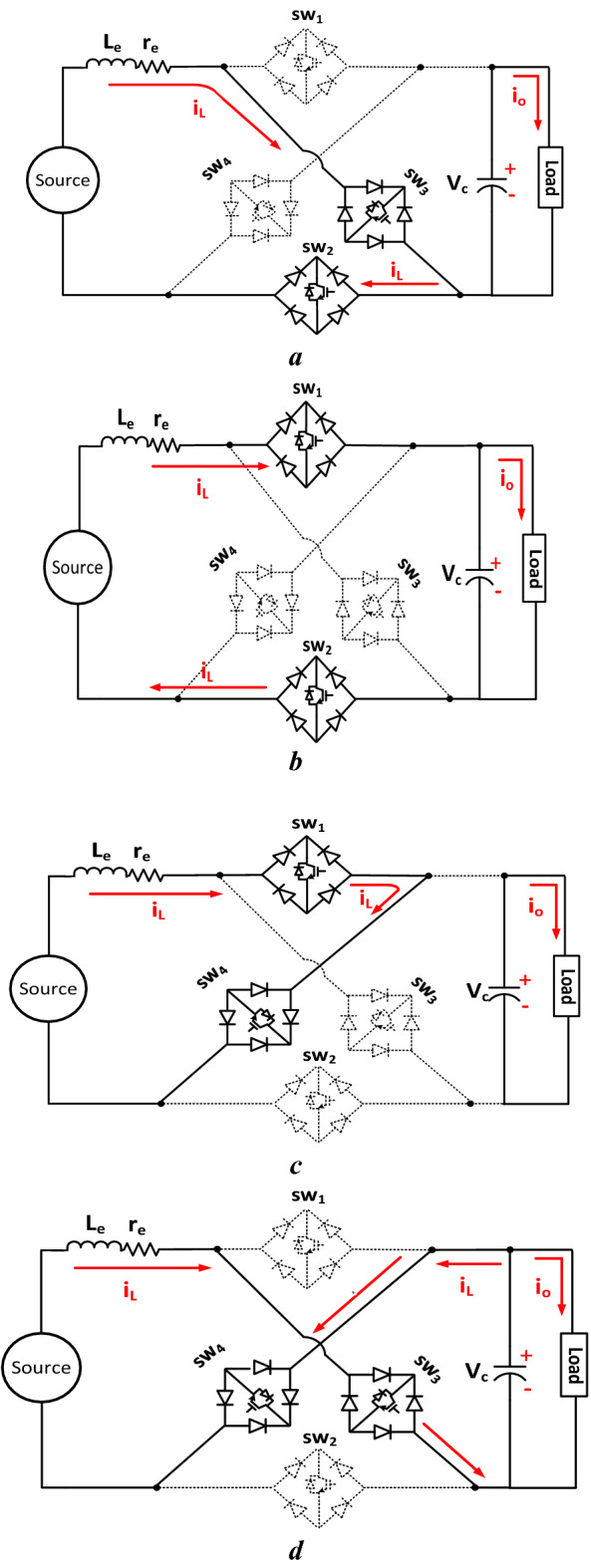
Circuit operation modes.

The circuit operation to achieve this function is divided into two cases based on the required state of the converter outputs: namely a positive boost (PB) and a negative boost (NB). The operation modes of the circuit during the PB and NB cases are shown graphically in Fig. 2, where the solid lines indicate the paths of the circuit currents during the various operation modes while the dotted lines refer to discontinuities in these currents.

The circuit operation as a DC–DC positive boost involves two modes as shown in Fig. 2a and b. In mode 1, the switches SW_3_ and SW_2_ are used to store energy in the inductor, *L*_*e*_, which is transferred to the load via SW_1_ and SW_2_ during mode 2. Also, the circuit operation as a negative boost involves two modes as shown graphically in Fig. 2c and d. In mode 1, SW_1_ and SW_4_ are switched on to energise the inductor while SW_3_ and SW_4_ are switched on to transfer the stored energy to the load and complete the operation cycle on the NB case. It may be stated that the sequence of the mentioned pairs of switches enables the proposed operation as a DC-DC converter, producing either a positive or negative output voltage.

The mathematical description of the above-mentioned operation modes can be written as follows:

#### Operation as DC–DC Positive Boost

**Mode 1,** Fig. 2a (switches SW_3_ and SW_2_ are on)

1 $$V_{in} - L_{e} \frac{{di_{l} }}{dt} - r_{e} {*}i_{l} = 0$$

2 $${ }v_{c} = i_{o} {*}R_{o} + L_{o} \frac{{di_{o} }}{dt}$$

3 $$\frac{{dv_{c} }}{dt} = \frac{{ - i_{o} }}{c}$$

**Mode 2,** Fig. 2b (switches SW_1_ and SW_2_ are on)

4 $$V_{in} - v_{c} - L_{e} \frac{{di_{l} }}{dt} - r_{e} {*}i_{l} = 0$$

5 $$v_{c} = i_{o} {*}R_{o} + L_{o} \frac{{di_{o} }}{dt}$$

6 $$\frac{{dv_{c} }}{dt} = \frac{{i_{l} - i_{o} }}{c}$$

#### Operation as DC–DC negative boost

**Mode 1,** Fig. 2c: The charging of the inductor is carried out in this mode via switching SW_1_ and SW_4_ on as shown in the figure. Therefore, the mathematical equations for this mode are similar to those used to represent Mode 1 of the PB case. Specifically, Eqs. (1–3) can be used to describe this mode of operation.

**Mode 2,** Fig. 2d, the cycle of the NB is completed via switches SW_3_ and SW_4_. The equations that describe this mode can be written as:

7 $$V_{in} + v_{c} - L_{e} \frac{{di_{l} }}{dt} - r_{e} {*}i_{l} = 0$$

8 $${ }v_{c} = i_{o} {*}R_{o} + L_{o} \frac{{di_{o} }}{dt}$$

9 $$\frac{{dv_{c} }}{dt} = \frac{{ - i_{l} - i_{o} }}{c}$$

### Circuit operation as a DC–AC boost converter

The operation principles of the proposed circuit as an inverter are achieved via four modes of operation to obtain the desired AC output. These operation modes are shown graphically in Fig. 2, where modes 1 and 2 result in the positive half-cycle while modes 3 and 4 complete the negative half-cycle.

The flow of the circuit currents and switches operating during the four modes is shown in Fig. 2.

**Mode 1,** Fig. 2a: This is considered the boosting mode of the converter circuit during the positive half-cycle where SW_3_ and SW_2_ are turned on. The energy is stored in the inductor, *L*_*e*_ as the inductor current increases, the load is fed from the energy stored in the capacitor.

**Mode 2,** Fig. 2b: This mode is considered the power conversion mode for the converter circuit during the positive half-cycle where SW_1_ and SW_2_ are turned on. The energy stored in the inductor, *L*_*e,*_ is transferred to the load and capacitor due to reversion of the voltage polarity across the inductor. A part of this stored energy is delivered to the load and the other part is used to charge the capacitor with a voltage level higher than the input DC voltage. This increases the capacitor voltage, *V*_*c*_, to the required boosting level.

The operation modes 1 and 2 for producing the positive half-cycle of the inverter output are similar to those used for the DC-DC positive boost. Therefore, Eqs. (1–3) can be used to represent mode 1 in the DC–AC converter, while Eqs. (4–6) can be used to describe mode 2 in the DC-AC converter.

**Mode 3,** Fig. 2c: In this mode, SW_1_ and SW_4_ are turned on in a similar way to that of Mode 1, negative boost. However, the equivalent circuit of this mode is similar to that of mode 1 when the circuit is operated as a DC–DC positive boost. Therefore, Eqs. (1–3) can be used to describe this operation mode.

**Mode 4,** Fig. 2d: Similarly, the equivalent circuit of this mode is similar to that of mode 2 of the operation, which is a DC-DC negative boost. Therefore, Eqs. (7–9) can be used to describe this mode.

### Circuit operation as an AC–AC boost converter

The circuit operation as an AC-AC boost converter involves only mode 1 and mode 2 is shown in Fig. 2, to obtain the desired AC output from the AC input. These modes can be described as follows to obtain the output positive half-cycle from the positive half-cycle of the input AC source:

**Mode 1,** Fig. 2a: This mode is used to energise the inductor via SW_3_ and SW_2_ switches. This leads to use Eqs. (1–3) to represent this mode.

**Mode 2,** Fig. 2b: This mode is used to transfer energy to the load via SW_1_ and SW_2_. This leads to the use of Eqs. (4–6) to represent this mode.

The above two modes are repeated to produce the negative half-cycle of the output as the input AC source becomes negative. Therefore, only the logic-based controller can be employed with modes 1 and 2 of Fig. 2 to operate the circuit as an AC-AC converter.

### Circuit operation as an AC–DC boost converter

The proposed circuit's operation as an AC-DC boost rectifier involves four modes to obtain the desired DC output. The circuit operation principles and modes are shown graphically in Fig. 2. The achievement of this function requires the sequence order of mode 1, mode 2, mode 3 and mode 4. Mode 1 is completed via switching on SW_3_ and SW_2_, and mode 3 occurs via SW_1_ and SW_4_. The equivalent circuit of both modes leads to employ Eqs. (1–3) to represent both modes. The equivalent circuit for mode 2 leads to use Eqs. (4–6) to represent this mode as it is completed via SW_1_ and SW_2_. Similarly, mode 4 is represented by Eqs. (7–9) as the mode requires setting on the switches SW_3_ and SW_4_.

The proposed circuit operation as a multi-function converter is summarized in the truth table given in Table 2, where '**1**' refers to the on-state of the switches while '**0**' represents the off-state.

**Table 2:** Truth table for the proposed multi-function converter operation.

Function	Operating mode	SW1	SW2	SW3	SW4
DC–DC boost positive	MODE 1	0	1	1	0
	MODE 2	1	1	0	0
DC–DC boost negative	MODE 1	1	0	0	1
	MODE 2	0	0	1	1
DC–AC boost	MODE 1	0	1	1	0
	MODE 2	1	1	0	0
	MODE 3	1	0	0	1
	MODE 4	0	0	1	1
AC–AC boost	MODE 1	0	1	1	0
	MODE 2	1	1	0	0
AC–DC boost	MODE 1	0	1	1	0
	MODE 2	1	1	0	0
	MODE 3	1	0	0	1
	MODE 4	0	0	1	1

## State-space stability analysis

### Time-invariant state-space model

The stable operation of the converter relies on the knowledge of its dynamic characteristics and the suitable application of feedback and compensation. This section illustrates the time-invariant state-space representation of the converter, demonstrates the stability analysis, and indicates the parameter effects and frequency response nature of the converter^32–34^. First, the state vector *X* is chosen as $$X = {( {i}_{L} {I}_{o} {v}_{c} )}^{t}$$ and the input vector *U* as *U* = ($${V}_{in})$$. The equations representing the DC-DC boost during the mode 1 can be written as:

10 $$\left[ {\begin{array}{*{20}c} {\frac{{\dot{d}i_{l} }}{dt}} \\ {\frac{{di_{o} }}{dt}} \\ {\frac{{dv_{c} }}{dt}} \\ \end{array} } \right] = \left[ {\begin{array}{*{20}c} { - { }\frac{{r_{e} }}{{L_{e} }}} & 0 & 0 \\ 0 & { - \frac{{R_{o} }}{{L_{o} }}} & {\frac{1}{{L_{o} }}} \\ 0 & { - \frac{1}{c}} & 0 \\ \end{array} } \right]\left[ {\begin{array}{*{20}c} {i_{l} } \\ {i_{o} } \\ {v_{c} } \\ \end{array} } \right] + \left[ {\begin{array}{*{20}c} {\frac{1}{{L_{e} }}} \\ 0 \\ 0 \\ \end{array} } \right]\left[ {V_{in} } \right]$$

In the standard state-space format, Eq. (10) can be written as follows:

11 $$\dot{X}\, = \;{ }A_{on} \cdot X + B_{on} \cdot { }U$$

Also, the equations representing mode 2 can be written as:

12 $$\left[ {\begin{array}{*{20}c} {\frac{{\dot{d}i_{l} }}{dt}} \\ {\frac{{di_{o} }}{dt}} \\ {\frac{{dv_{c} }}{dt}} \\ \end{array} } \right] = \left[ {\begin{array}{*{20}c} { - { }\frac{{r_{e} }}{{L_{e} }}} & 0 & { - \frac{1}{{L_{e} }}} \\ 0 & { - \frac{{R_{o} }}{{L_{o} }}} & {\frac{1}{{L_{o} }}} \\ \frac{1}{c} & { - \frac{1}{c}} & 0 \\ \end{array} } \right]\left[ {\begin{array}{*{20}c} {i_{l} } \\ {i_{o} } \\ {v_{c} } \\ \end{array} } \right] + \left[ {\begin{array}{*{20}c} {\frac{1}{{L_{e} }}} \\ 0 \\ 0 \\ \end{array} } \right]\left[ {V_{in} } \right]$$

In the standard state-space format, Eq. (12) can be written as follows:

13 $$\dot{X}\; = \;A_{off} \cdot X + B_{off} \cdot U$$

Assuming the ON-period duration is *D*, the OFF-period duration will be 1 $-$ *D* to represent one cycle. Therefore, using the average technique^32–34^, the overall state-space model of the converter can be written as:

14 $$\dot{X} = { }\left[ {D \cdot { }A_{on} + \left( {1 - D} \right){ } \cdot { }A_{off} } \right] \cdot { }X + { }\left[ {D \cdot { }B_{on} + \left( {1 - D} \right){ } \cdot { }B_{off} } \right]{ } \cdot { }U$$

Perturbing Eq. (14) about a steady-state point where the state vector *X* = *X*_0_ + *x* and *D* = *D*_*0*_ + *d* yields;

15 $$\dot{X}_{0} + { }\dot{x} = { }\left[ {\left( {D_{0} + d} \right) \cdot A_{on} + \left( {1 - D_{0} - d} \right) \cdot A_{off} } \right] \cdot { }\left( {{ }X_{0} + x} \right) + \left[ {\left( {D_{0} + d} \right) \cdot B_{on} + \left( {1 - D_{0} - d} \right) \cdot B_{off} } \right] \cdot U{ }$$

Equation (15) can be expanding to

16 $$\begin{aligned} \dot{X}_{0} + { }\dot{x} & = \left[ {D_{0} { }A_{on} + \left( {1 - D_{0} } \right){ } \cdot A_{off} } \right] \cdot X_{0} \\ & \;\; + \left[ {D_{0} \cdot B_{on} + \left( {1 - D_{0} } \right){ } \cdot B_{off} } \right] \cdot U \\ & \;\;{ } + { }\left[ {d \cdot A_{on} - d \cdot A_{off} } \right] \cdot X_{0} { } + \left[ {d \cdot B_{on} - d \cdot B_{off} } \right]{ } \cdot { }U \\ { } & \;\; + { }\left[ {D_{0} \cdot A_{on} + \left( {1 - D_{0} } \right) \cdot A_{off} } \right] \cdot { }x{ } \\ & \;\;{ } + \left[ {{ }d \cdot A_{on} - d \cdot A_{off} } \right] \cdot { }x \\ \end{aligned}$$

Subtracting Eq. (14) from Eq. (16) and neglecting the higher order deviations yields:

17 $$\dot{X} = A . x + F . d$$

where : $$A = \;{ }D_{0} A_{on} + \left( {{ }1 - D_{0} } \right)A_{off} { }$$

$$F\; = \;\left[ {{ }A_{on} - { }A_{off} { }} \right]{ } \cdot { }X_{0} + { }\left[ {B_{on} - { }B_{off} { }} \right]{ } \cdot U$$

Equation (17) can be solved using the Laplace transform to obtain the converter transfer function as:

18 $${ }\frac{{{ }x}}{d}{ } = \left[ {sI - A} \right]^{ - 1} F$$

19 $${ }\frac{{{ }x}}{d}{ } = \frac{{\left[ {\begin{array}{*{20}c} {\widehat{{i_{l} }}} \\ {\widehat{{i_{o} }}} \\ {\widehat{{v_{c} }}} \\ \end{array} } \right]}}{d} = \frac{{adj{ }\left[ {sI - A{ }} \right]F}}{{{\text{det}}\left[ {{\text{SI}} - {\text{A }}} \right]}}$$

A Matlab program is built, using a Matlab symbolic reduction method, to obtain the transfer function displayed in Eq. (19). Neglecting the inductor resistance, the converter transfer function is obtained as:

20 $$\frac{vc}{d} = \frac{{V_{in} }}{{ \left( {1 - D} \right)^{2} }} \frac{{ - { }L_{e} {\text{ S}} + { }R_{o} \left( {1 - D} \right)^{2} }}{{R_{o} { }L_{e} C S^{2} + { }L_{e} {\text{ S }} + R_{o} \left( {1 - D} \right)^{2} { }}}$$

Equation (20) can be written in the standard transfer function pole-zero format:

21 $$\frac{{ {\mathbf{V}}_{{\mathbf{C}}} }}{{\mathbf{d}}}\;{ } = \;{\text{G}}_{0} \;\;\;{ }\frac{{1 - \frac{S}{{\tau_{z} }}}}{{\frac{{{\text{S}}^{2} }}{{{\text{w}}_{0}^{2} }}\; + \;{ }\frac{{\text{S}}}{{{\text{Qw}}_{0} }}{ }\; + \;{ }1}}$$

where $G_{0} = \;steady\; state\; gain\; = \frac{{V_{in} }}{{\left( {1 - D} \right)^{2} }}$, $w_{o} = Natural\,\, frequency = \frac{1 - D}{{\sqrt {L_{e} C} }}$, $Q \; = \; quality\; factor \; = \;R_{o} \left( { 1 - D } \right) \sqrt {\frac{C}{{L_{e} }} }$; $\tau_{Z} \; = \; \frac{{R_{o} \left( {1 - D} \right)^{2} }}{{L_{e} }}$, where $\tau_{Z}$ is a time constant.

Equation (21) illustrates that the converter has one zero at *zero* in Right-Hand Side (RHS) of the *S*-plane and two complex conjugate poles at the Left-Hand Side (LHS) of the *S*-plane. The zero is at S = $\tau_{Z}$ while the poles are located at:

22 $$S_{1, 2} = \frac{{W_{o} }}{2 Q} \left( { - 1 \pm \sqrt {1 - 4 Q^{2 } } } \right)$$

The presence of the zero on the RHS, Eq. (21), indicates that the converter follows the characteristics of the non-minimum phase systems, which requires careful stability analysis as the stability analysis using the phase and gain margins only leads to misleading results^35,36^. Therefore, the effects of the converter parameters are explained in terms of Pole-Zero and Bode-Plot diagrams in the following section. This study is carried out at capacitance, C, equals 5, 50 and 150 µF while the values of the inductance ${L}_{e}$ equals 5, 15 and 75 mH.

### Converter design

Designing the converter represents a crucial issue since the parameter values, switching frequency, and the rating of the components are critically needed to be determined. The criteria for parameter estimation can be found in^4^ and can be summarized and linked to the proposed converter as follows: For the boost DC-DC converter, the input current ripples and the output voltage ripples and be acquired from the following equations.

23 $${\Delta }I = \frac{{V_{in} *D}}{{f_{sw} *L_{e} }}$$

24 $${\Delta }V_{o} = \frac{{\left( {I_{L} - I_{0} } \right)*D}}{{f_{sw} *C}}$$

where $\Delta I$ is the input current ripple, $\Delta {V}_{o}$ is the output voltage ripple, ${V}_{in}$ is the supply voltage, $D$ is the duty ratio, ${f}_{sw}$ is the switching frequency, ${I}_{L}$ is the average value of the inductor current, ${I}_{0}$ is the average value of the load current, ${L}_{e}$ is the input inductance, and $C$ is the output capacitance. As illustrated in Eqs. (23) and (24), the switching frequency, input inductance, and output capacitance are related to each other and to the ripples in the output voltage and input current. So, it is required to choose the values of them in order to obtaining suitable values for the ripples. Firstly, the switching frequency is chosen based on the switching rating of the switches and the sampling rate of the control board, and the switching losses can be calculated from the following equation for Insulated Gate Bipolar Transistor (IGBT) switch.

25 $$P_{{l\left( {sw} \right)}} = \frac{{f_{sw} }}{6}{*}\left( {i_{c\left( s \right)*} v_{{ce\left( {cf} \right)}} \left( {t_{r} + t_{f} } \right)} \right)$$

where ${P}_{l\left(sw\right)}$ is the switching losses, ${i}_{c\left(s\right)}$ is saturation collector current, ${v}_{ce\left(cf\right)}$ is the cut-off value of the collector to emitter voltage, ${t}_{r}$ is the rising time of the switch, and ${t}_{f}$ is the falling time of the switch. Equation (25) is derived under many assumptions as illustrated at the Appendix. Then the capacitance and the inductance are chosen based on the selected switching frequency, load current, supply current, and the acceptable ripples in the load voltage and the supply currents. As seen from this design procedure documented in^4^, there is a variety in choosing the input inductance, output capacitor, and the switching frequency based on many other factors.

If the function of the converter is switched to be an inverter, then, the output capacitance is chosen in order to filter high order harmonics in the output voltage. In order to achieve that the capacitor is designed to make its capacitive reactance much lower than the load impedance for the harmonicas that are higher than fundamental one. While the inductance is designed to limit the input current ripples within suitable values as in Eq. (23).

For the rectifier operation. The capacitor design follows the same design steps for the boost converter in reducing the output voltage ripples, while the inductance is selected in such way that it works as an input current filter. This could be acquired by selecting a value that make the inductive reactance much high as possible with the higher order harmonics. When AC–AC function takes its turn, the output capacitor design follows that of the inverter. Furthermore, the inductance value is estimated as in the rectifier function.

As elaborated from the above design criteria. It is obvious that for each function there is a range for each parameter to select from it to obtain the appropriate performance of the converter. So, to make the converter works as a multifunction one, it would be compulsory to select values that are suitable for all functions, loads requirements, and the used switching frequency.

### Effects of converter parameters

**Figure 3:**
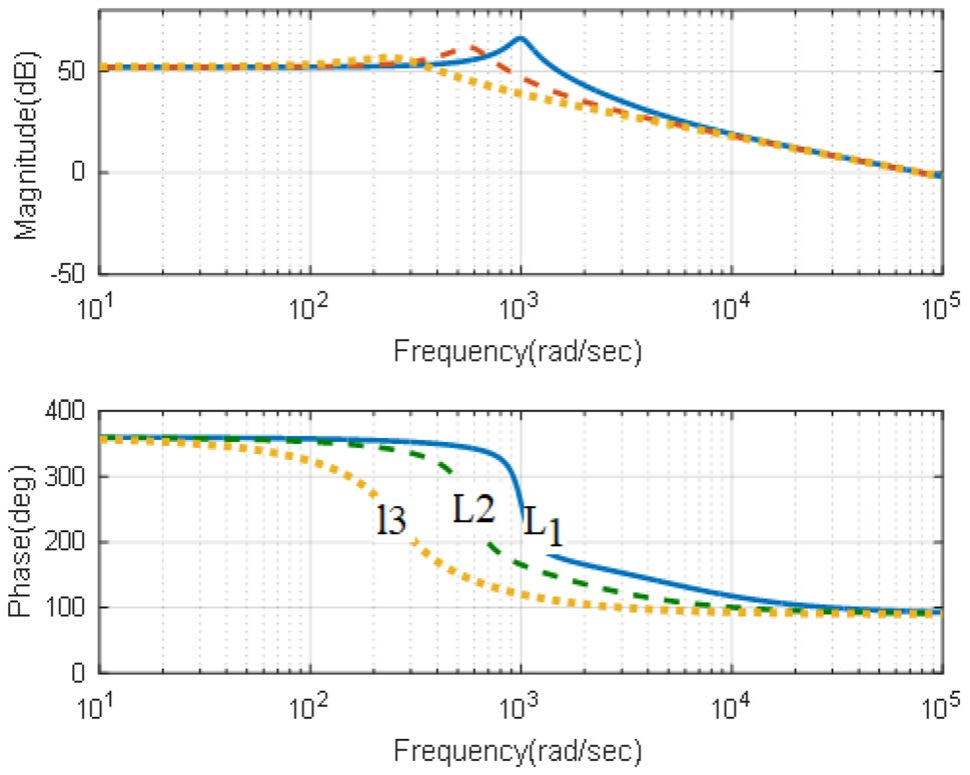
Effects of varying the inductance, Bode-plot.

**Figure 4:**
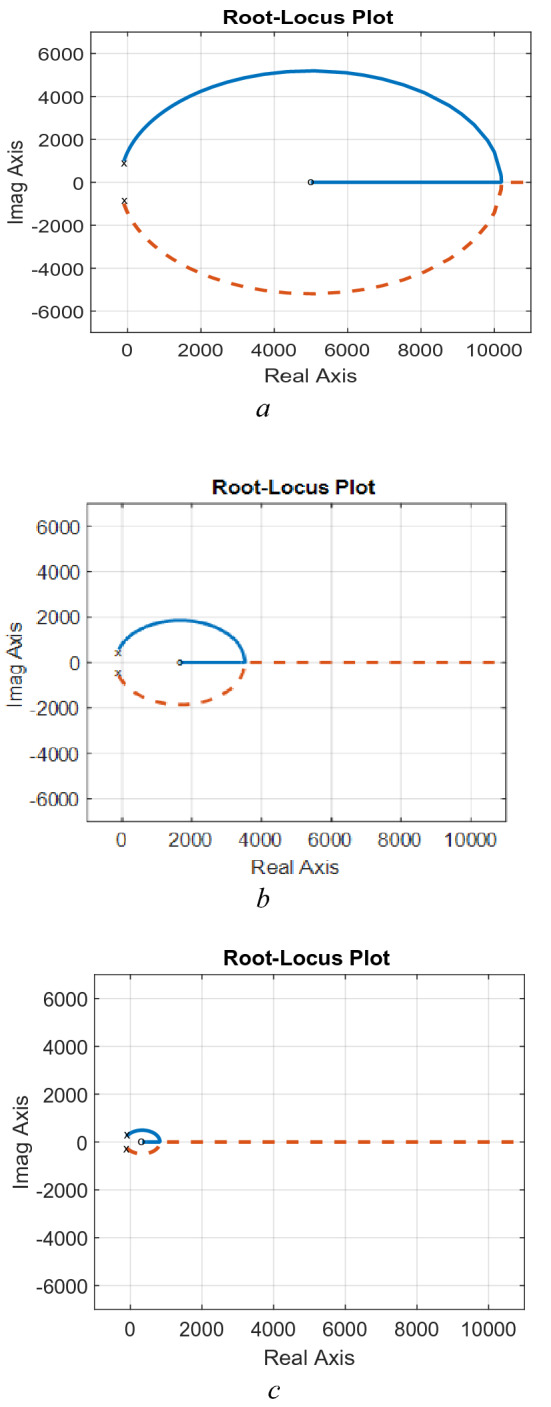
Effects of varying the inductance, Pole-Zero. (**a**) L = 5 mH, C = 50 µF, (**b**) L = 15 mH, C = 50 µF, (**c**) L = 75 mH, C = 50 µF.

Based on the design criteria elaborated in section “Converter design”, two studies can be obtained. The first one is obtaining the effects of varying the converter inductance at a constant value of the capacitance. It is obtained in terms of Bode-Plot and Pole-Zero diagrams are shown in Figs. 3 and 4. The Bode-Plot, Fig. 3, illustrates steady gain operation at low frequencies and − 40 dB/decade at higher frequencies that requires careful compensation to ensure adequate transient characteristics, which necessitate − 20 dB/decade. Furthermore, the Pole-zero diagram, Fig. 4, show that increasing the inductance has significant effects on the locations of the zero and negligible effects on locations of the converter poles as mentioned in Eq. (21).

**Figure 5:**
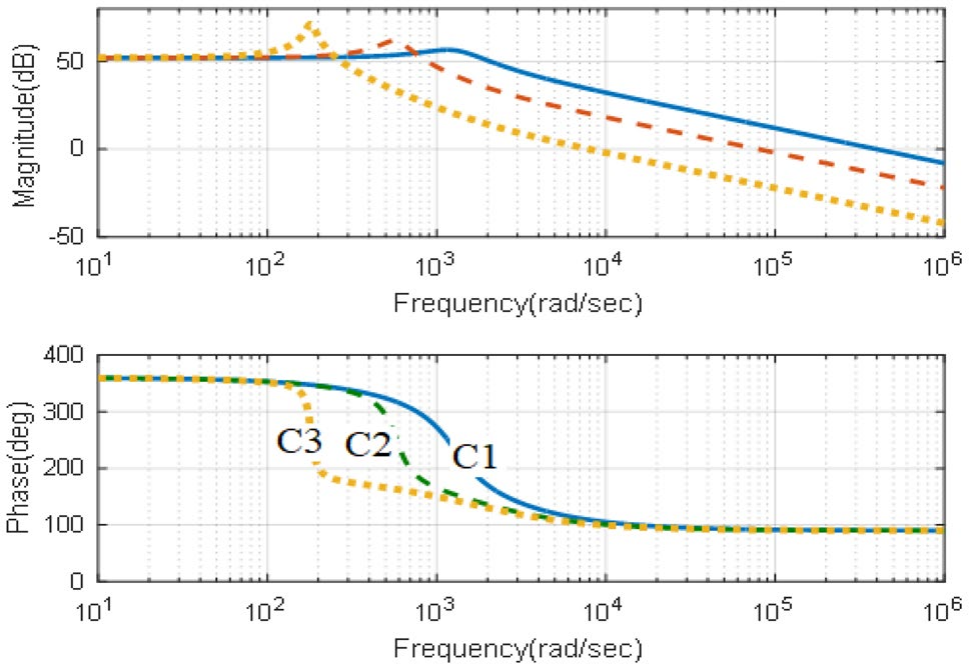
Effects of varying the inductance, Bode-plot.

**Figure 6:**
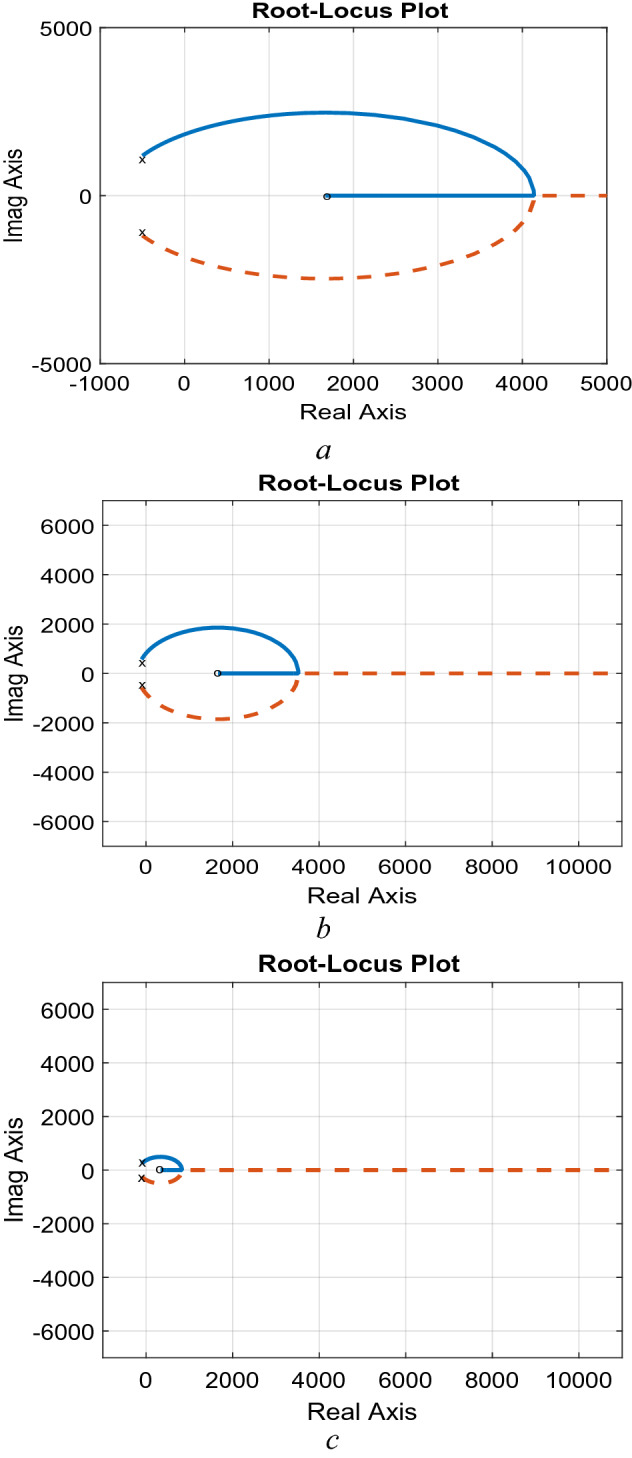
Effects of varying the capacitance, Pole-Zero. (**a**) L = 15 mH, C = 5 μF, (**b**) L = 15 mH, C = 50 μF, (**c**) L = 15 mH, C = 150 μF.

The second study is obtaining the effects of varying the converter capacitance, at a constant value of the inductance in terms of Bode-Plot and Pole-Zero diagrams are shown in Figs. 5 and 6. The Bode-Plot, Fig. 5, illustrates that varying the capacitance leads to similar characteristics to that obtained when varying the inductance. However, the most significant effects appear in the Pole-Zero diagram, Fig. 6, where varying the capacitance has a significant effect on the locations of poles of Eq. (21). The results illustrate that increasing the capacitances moves the pole locations towards the RHS of the S-plane, indicating sever effects on stability margins.

### System response

**Figure 7:**
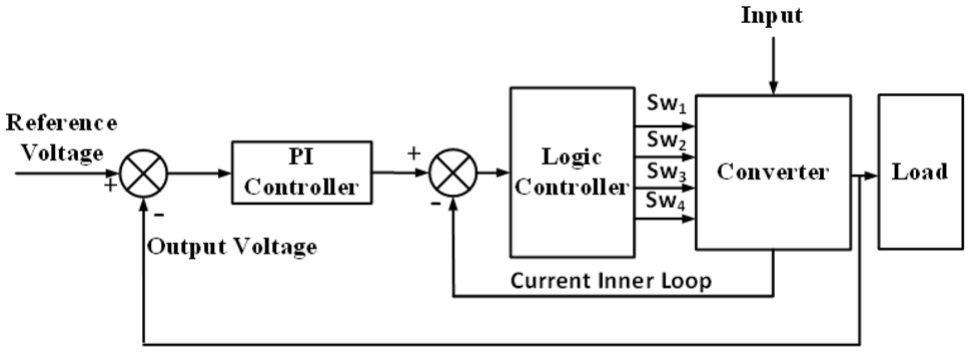
Implementation block diagram.

**Figure 8:**
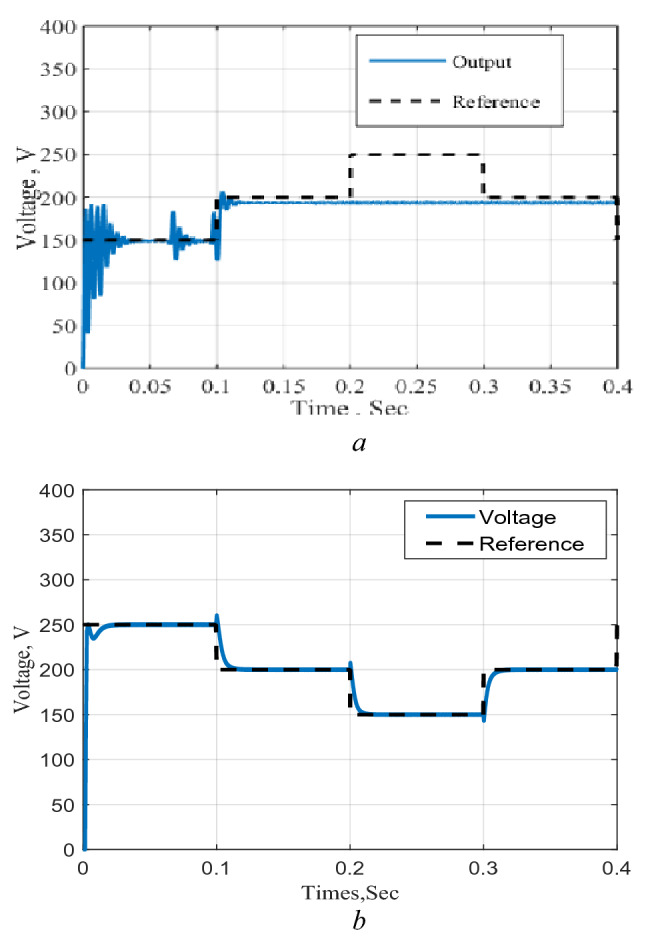
Response to successive changes in the reference voltage. (**a**) System response without inner loop system, (**b**) response with inner loop.

To confirm the remark obtained from the frequency domain analysis, the proposed circuit is simulated along with the logic-based control scheme using the Matlab/SimPower environments. The system is operated in closed loop with inner loop which employs a signal obtained from the inductor current as shown in Fig. 7. The system response to successive changes in the reference voltage and the results obtained for the two cases are shown in Fig. 8a and b where the dotted lines represent the reference value, and the solid lines are the converter output voltage. The results show that the system response with the inner loop, Fig. 8a, follows any changes in the required output reference voltage successfully over a wide range of the boosting levels. However, this is not achieved for the system without the inner loop, Fig. 8b, due to the presence of a *zero* at the RHS of the S-plane.

## Simulation results

The proposed circuit performance is obtained and evaluated when operated as a multi-function converter as will be described subsequently. Tables 3 and 4 introduce the converter specifications and control parameters, respectively.

**Table 3:** Converter specifications.

Symbol	Meaning	Value
${{\varvec{V}}}_{{\varvec{i}}{\varvec{n}}}$	Input voltage	100 V max
**L**_**e**_	Coil inductance	15 mH
**C**	Output capacitance	50 $\upmu F$
${{\varvec{r}}}_{{\varvec{e}}}$	Inductor internal resistance	1 Ω
${{\varvec{R}}}_{{\varvec{o}}}$	Output resistance	100 ohms
${{\varvec{L}}}_{{\varvec{o}}}$	Output inductance	100 mH

**Table 4:** Converter controller gains.

Function	K_p_	K_i_
DC-DC	0.02	12
DC-AC	0.005	1.85
AC-AC	0.1	1.5
AC-DC	0.01	5

### Circuit performance as a DC–DC boost converter

**Figure 9:**
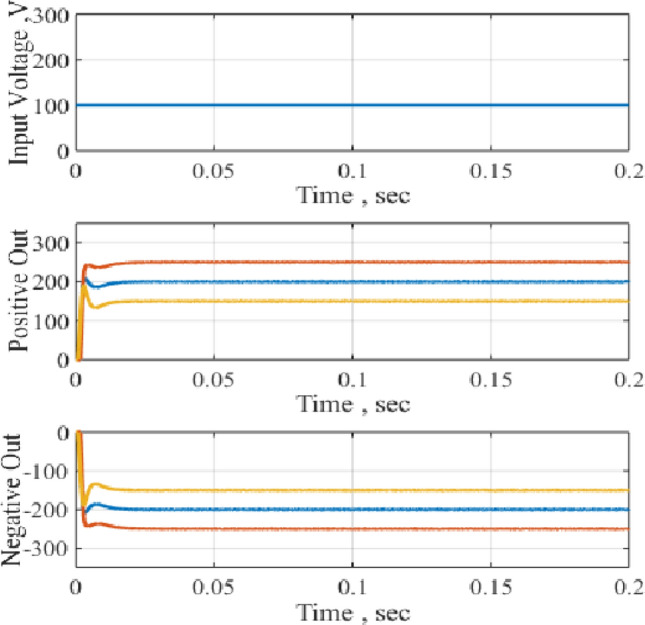
Response to 150%, 200% and 250% of supply voltage, DC–DC.

The circuit performance when operated as a DC-DC converter is obtained as shown in Fig. 9. This is the response of the DC-DC positive boost to various changes in the boosting level, namely, 150%, 200% and 250% while the input voltage remains 100 V as shown in Fig. 9. Similar results are obtained for the DC–DC negative boost and the results are shown in Fig. 9. The results illustrate precise control of the boosting level with minimum overshoot during the operation of the circuit as a DC-DC positive and DC-DC negative boost.

### Performance of the DC–AC boost function

**Figure 10:**
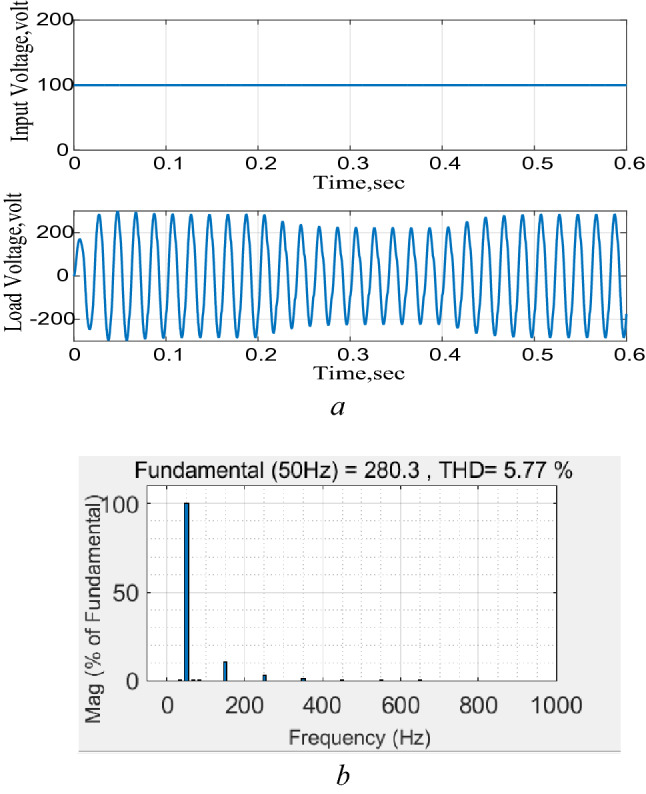
Response to a step change in the reference voltage. (**a**) DC Input voltage and output AC voltage, (**b**) total harmonic distortion (THD).

The system is connected to a 100 V DC supply and subjected to a 25% change in the voltage reference level for 200 ms and the results are shown in Fig. 10. The results show a good performance and successful operation of the proposed circuit as an inverter with the capability of following any changes in the output reference. The FFT analysis also shows that the total harmonic distortion (THD) of the output voltage waveform is less than 6% which represents an acceptable level as shown in Fig. 10b.

### Circuit performance as an AC–AC boost converter

**Figure 11:**
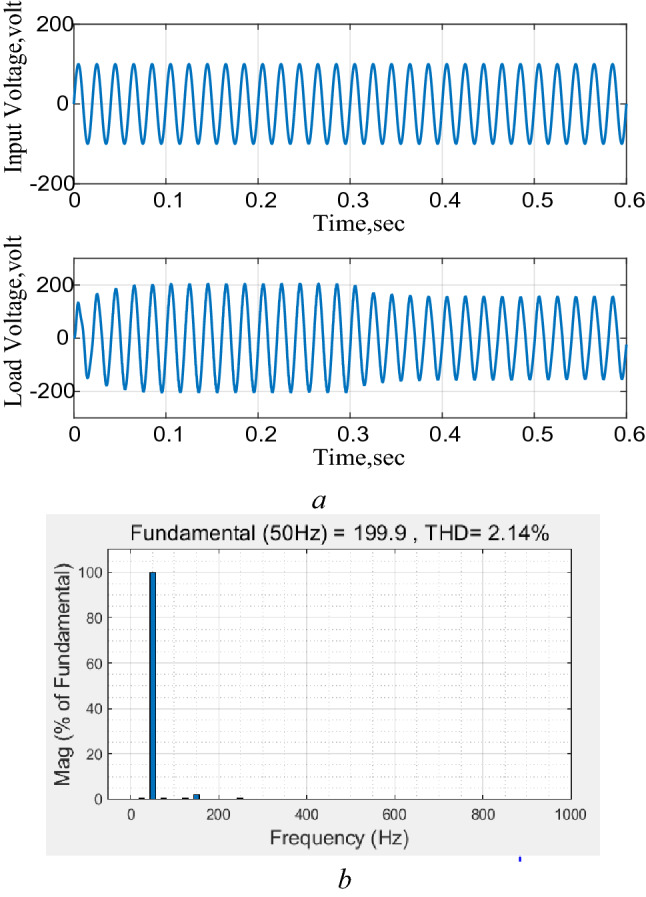
Response to a 25% step reduction in the reference voltage, AC–AC. (**a**) Input and output voltages, (**b**) total harmonic distortion (THD).

The converter is connected to a 100 V AC supply followed by a 25% step reduction in the reference output voltage applied at *t* = 0.3 s and the results are shown in Fig. 11. The results show an excellent performance with almost pure sinusoidal output as the level of THD is about 2% as shown in Fig. 11b. The results also illustrate that the converter output follows any changes in the reference output voltage.

### Circuit performance as an AC–DC boost converter

**Figure 12:**
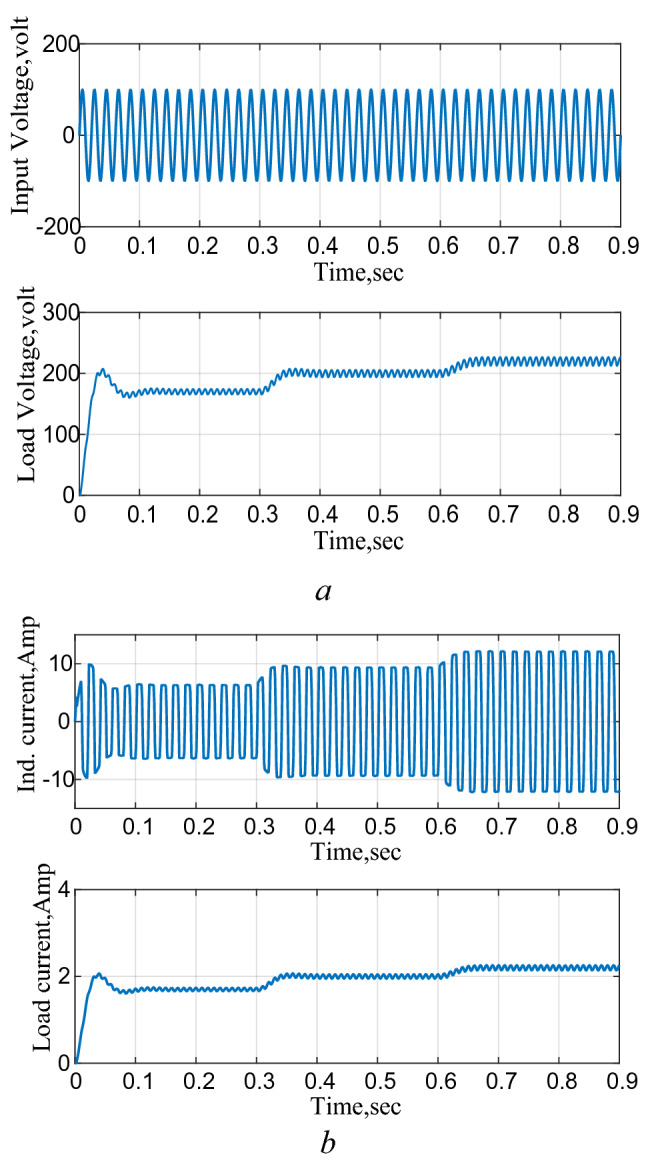
Response to a successive change in the reference voltage, AC–DC. (**a**) Input/output voltages, (**b**) inductor and load currents.

The circuit is then connected to a 100 V AC supply and subjected to successive changes in reference DC output voltage and the results are shown in Fig. 12. These results also demonstrate the capability of the proposed circuit to operate as a rectifier and successfully follows any changes in the required output with less than 4% ripple in the output DC voltage.

## Experimental setup

To demonstrate the physical realization and real-time implementation of the proposed circuit as a MFC, a DSP-based laboratory model is built in the laboratory using MPC 8240 digital signal processor dSPACE (DS-1104) with PPC 603e core. The processor is clocked at 250 MHz, including hardware interface components, software, and a measurements kit. The dSPACE (DS-1104) offers flexible and powerful tool for real-time implementation of control algorithms, enabling an excellent computer interface with digital to analogue (D/A) and analogue to digital (A/D) conversion capabilities. Each bridge-based bi-directional switch is built using an IGBT modules type MITSUBISHI CM100DY-24H and four fast-acting diodes. The switches are arranged on an isolated board with their snubbed circuits for each IGBT, the snubber circuits are designed based on the criteria found in^4^. The drive circuit that produces the required signals to drive the IGBTs and a signal transducer’s kits are built to measure the circuit current via current transducer LA25-NP and the circuit voltage via voltage transducer LV25-P. The laboratory model is used to test the performance of the proposed circuit when operating as a multi-function converter. The circuit is extensively tested, and a sample of the results are presented subsequently, demonstrating the physical realization of the proposed circuit topology that might aid recent developments in micro and smart power system grids and other applications.

**Figure 13:**
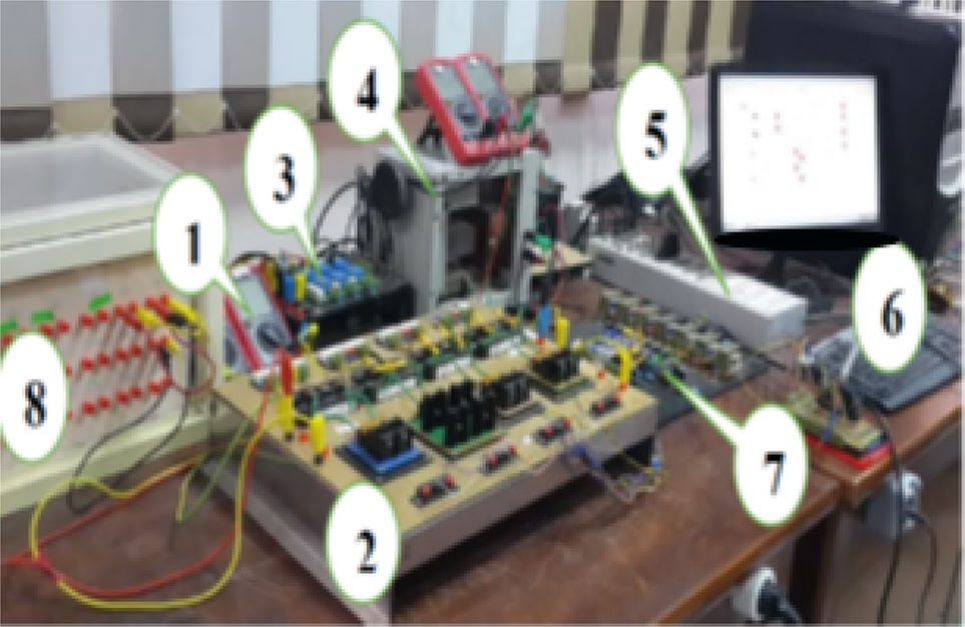
Experimental setup for the proposed circuit.

The real-time photo of the implementation of the proposed circuit is shown in Fig. 13 with the components listed in Table 5.

**Table 5:** Experimental components.

No.	Component	No.	Component
1	Measurements	5	DSP interface
2	MFC	6	Computer
3	Transducer Kit	7	Basic driving Circuit
4	Inductances Box	8	Capacitance Box

### Circuit performance as a DC–DC boost converter

**Figure 14:**
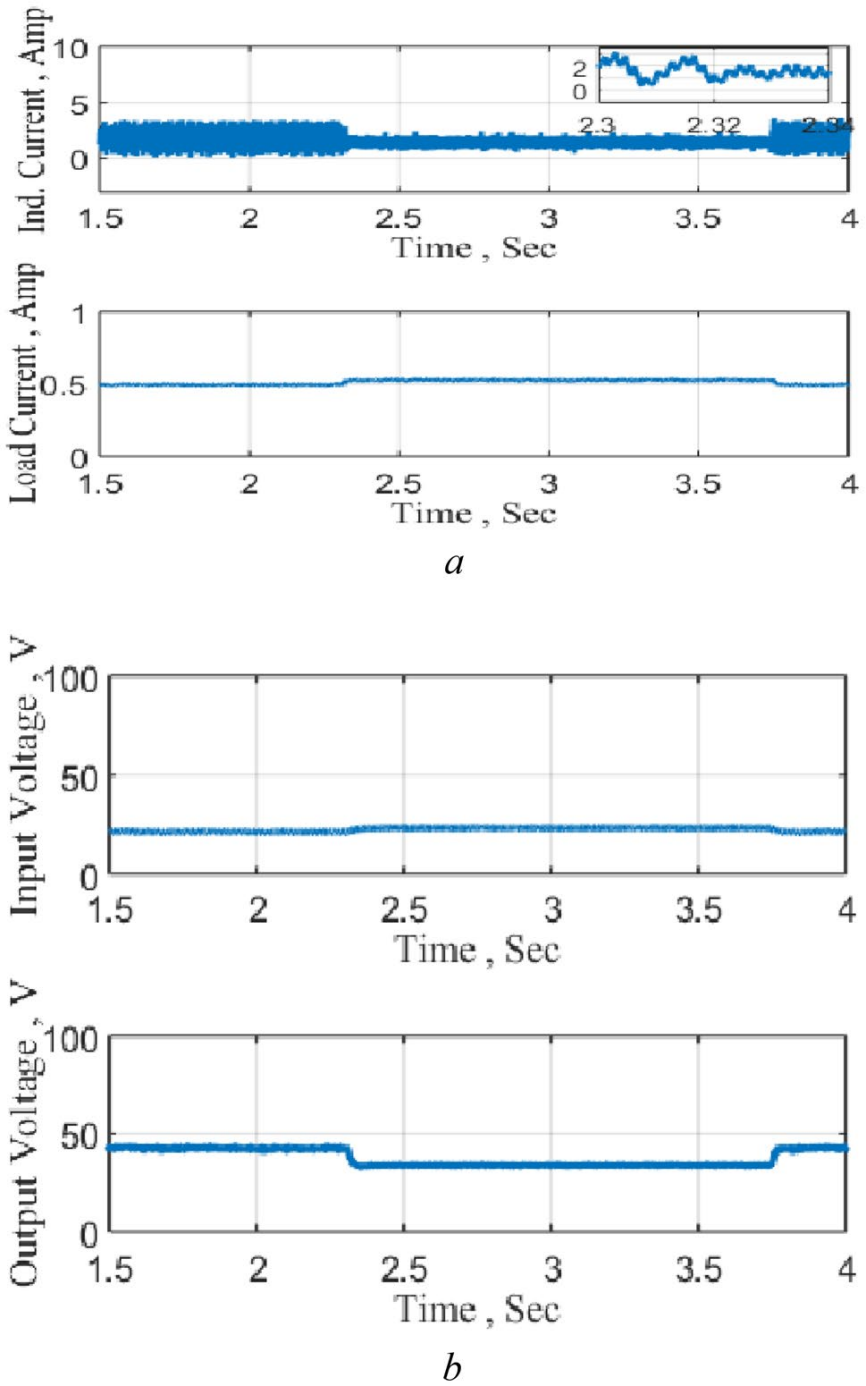
Circuit Performance as DC-DC converter, PB. (**a**) Inductor and output currents, (**b**) input and output voltages.

The converter is extensively tested as a DC-DC boost and a sample of the results is presented. Figure 14 shows the circuit performance when connected to a DC source and subjected to a step reduction in the reference voltage for about one and a half second. The results illustrate that the converter boosts the input voltage to the required boosting level and provide precise control during both the step reduction and increase in the reference level. The results also show the increase and decrease of the inductor current during the different modes of operation. It may be stated that the input voltage in Fig. 14 was measured at the switch SW_1_ terminal which justifies the increase in the input voltage due to the inductance effects due to the charging and discharging operation modes.

**Figure 15:**
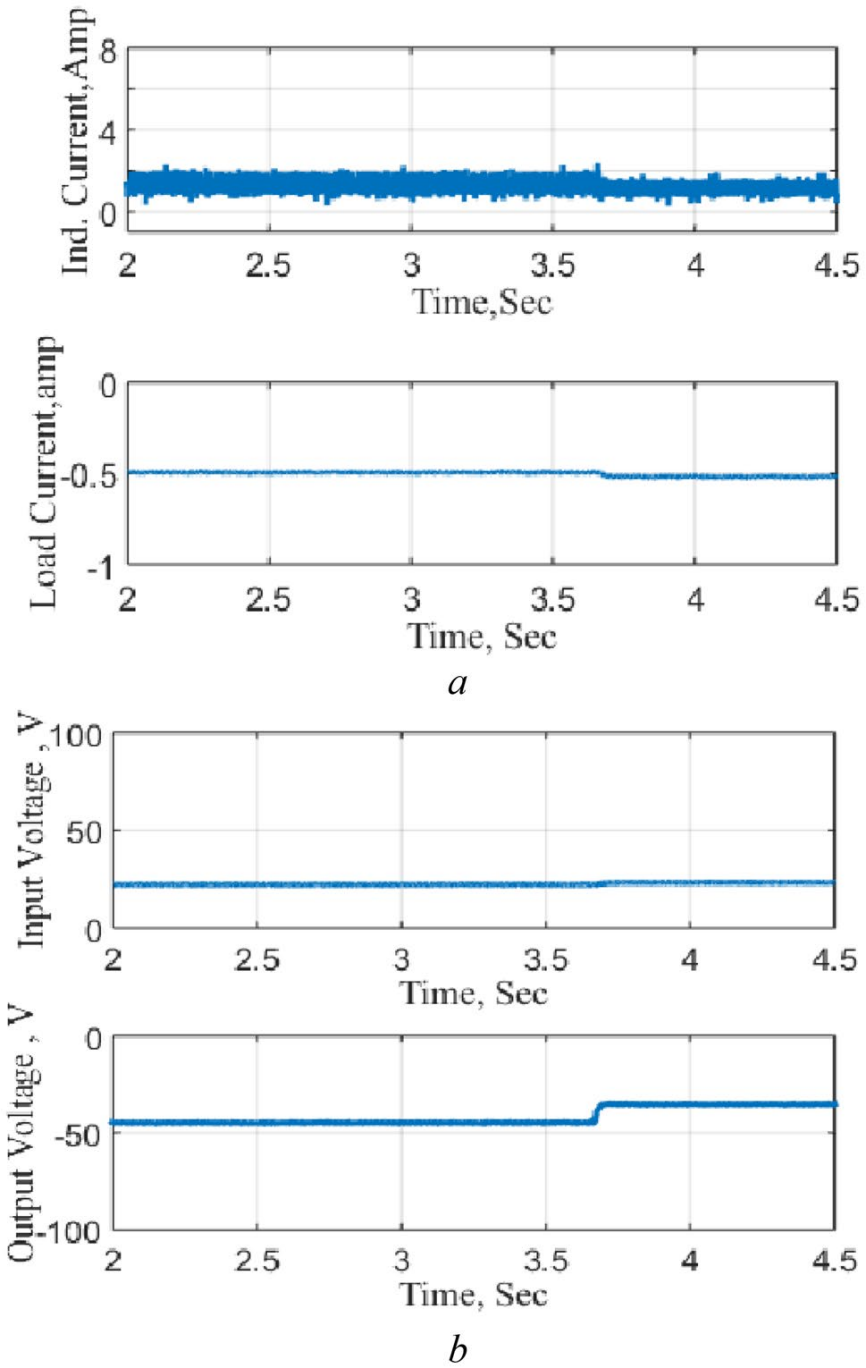
Response to a step decrease, NB. (**a**) Inductor and output currents, (**b**) input and output voltages.

The circuit is then tested as a negative boost when connected to the DC source and subjected to a step increase in the reference voltage and the results are shown in Fig. 15. The results illustrate the precise control of the output voltage without any overshoot following the change in the reference voltage. The results also clearly show the charging and discharging of the inductor current during the operation modes.

### Circuit performance as DC–AC converter

**Figure 16:**
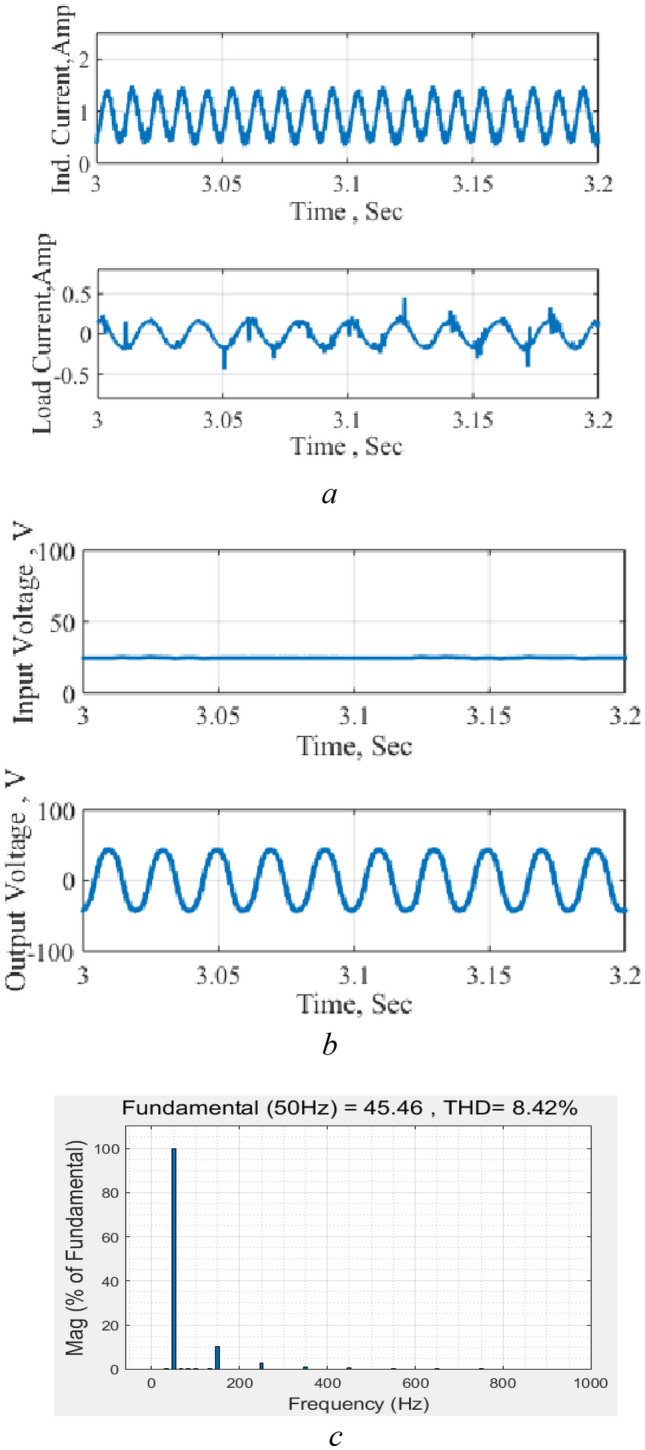
Circuit performance as an inverter. (**a**) Inductor and output current, (**b**) input and output voltages, (**c**) THD level.

The circuit response as a DC–AC converter is shown in Fig. 16. It may be stated that the input voltage is obtained in this test via a rectified single-phase bridge that justifies the ripples in the input voltage. The results show the good performance of the circuit as an inverter and maintain the good boosting capability. The THD is about 8% which is higher than the obtained by computer simulation due to distortion in measurements and ripples in the input source. However, the results encourage the implementation as an inverter in real power systems where the input may be via pure DC sources such as the photovoltaic or storage sources.

**Figure 17:**
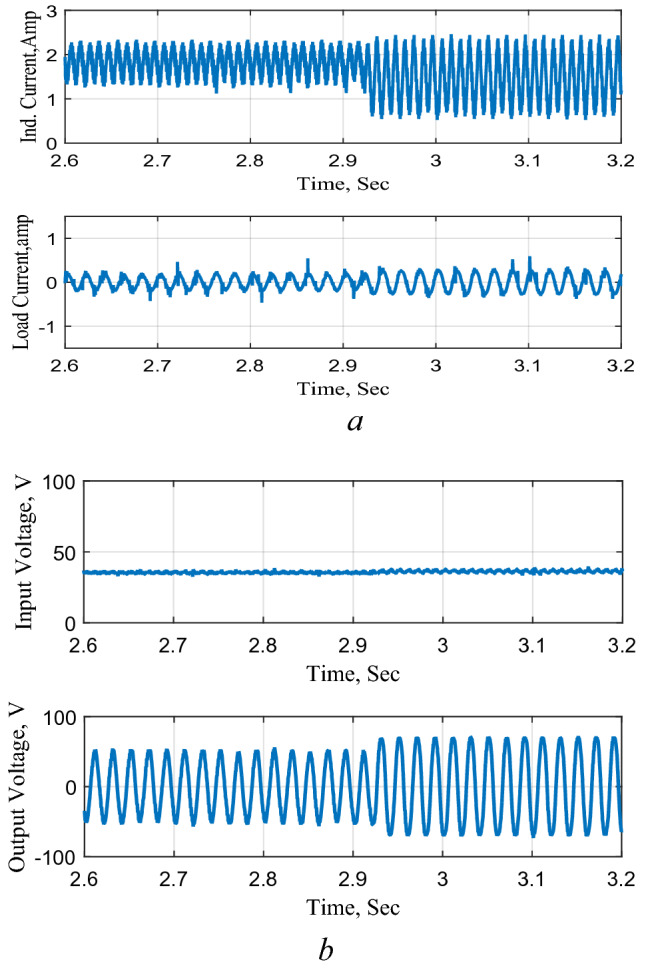
Response to a step change in the reference voltage. (**a**) Inductor and output currents, (**b**) input and output voltages.

Moreover, the circuit response to a step increase in the reference voltage is shown in Fig. 17 that confirms the successful operation of the circuit as an inverter under changing in the required output value and boosting level.

### Circuit performance as AC–AC converter

**Figure 18:**
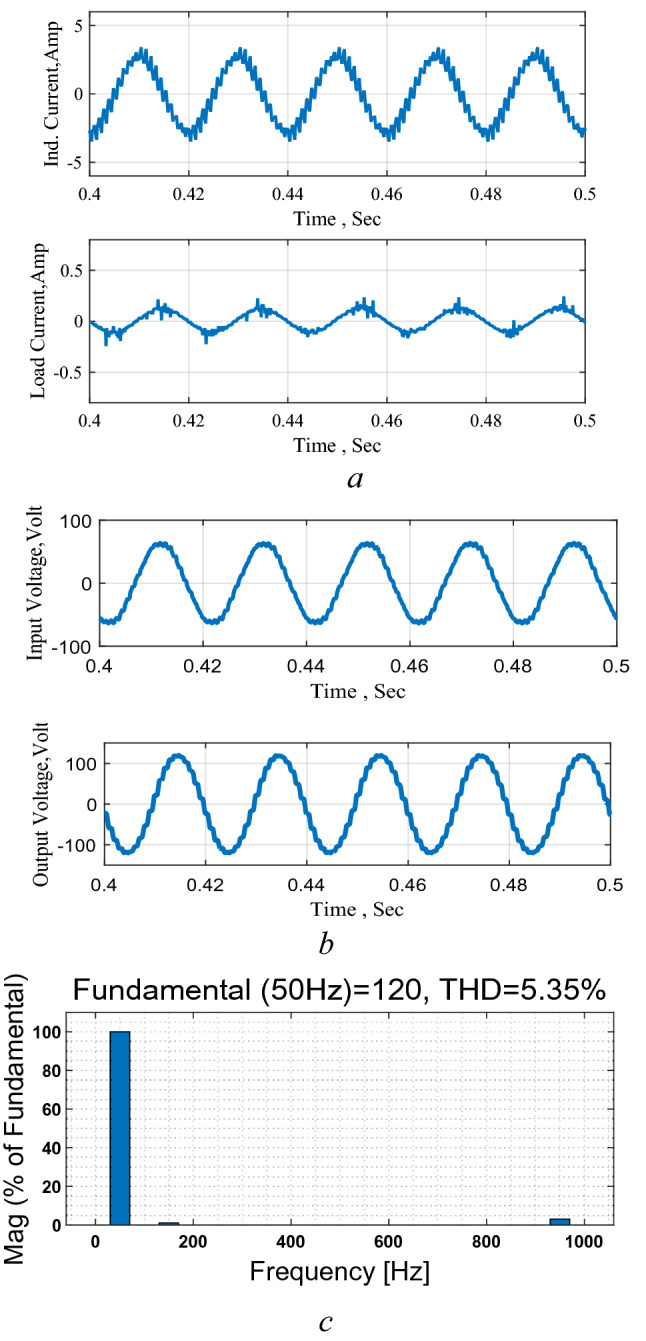
Circuit performance as an AC–AC converter. (**a**) Inductor and output currents, (**b**) input and output voltages, (**c**) THD Level.

Figure 18 shows the circuit performance as an AC-AC converter. The results illustrate good performance with an acceptable THD level (about 5%) as may be observed from Fig. 18c.

**Figure 19:**
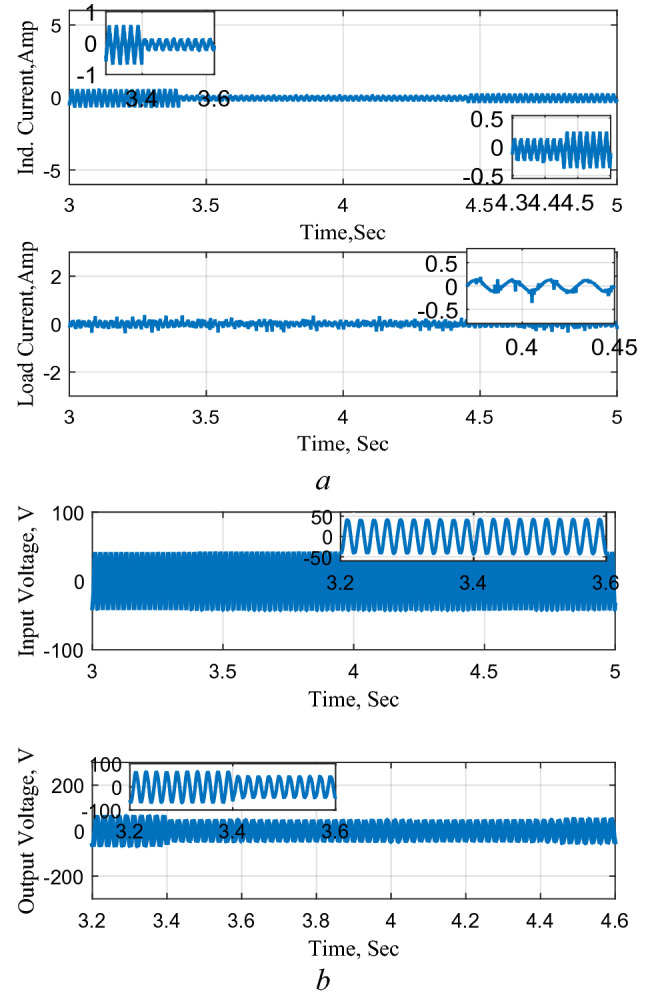
Response to a step change in the reference voltage. (**a**) Inductor and output currents, (**b**) input and output voltages.

Furthermore, the system is subjected to a step decrease and a step increase in the reference voltage and the results are shown in Fig. 19. The results also confirm the good operation of the circuit as AC–AC converter under changes in the required reference voltage.

### Circuit performance as AC–DC converter

**Figure 20:**
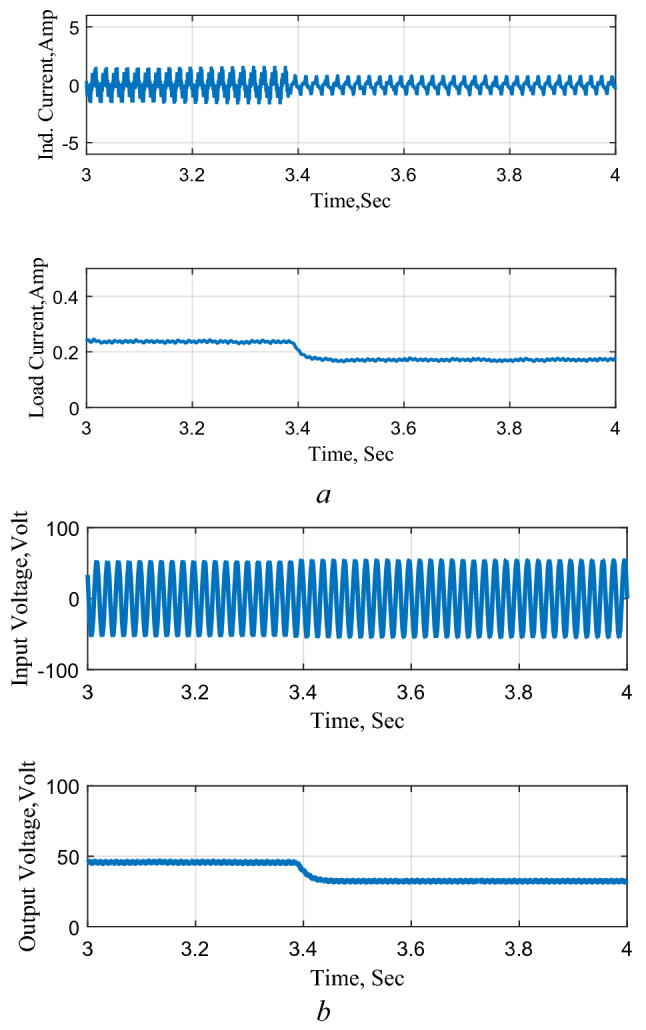
Response to a step change in the reference voltage AC–DC. (**a**) Inductor and output currents, (**b**) input and output voltages.

The circuit performance when operating as a rectifier is shown in Fig. 20, that shows the response to a step reduction in the reference voltage. This results also illustrate good performance and precise control of the output voltage.

**Figure 21:**
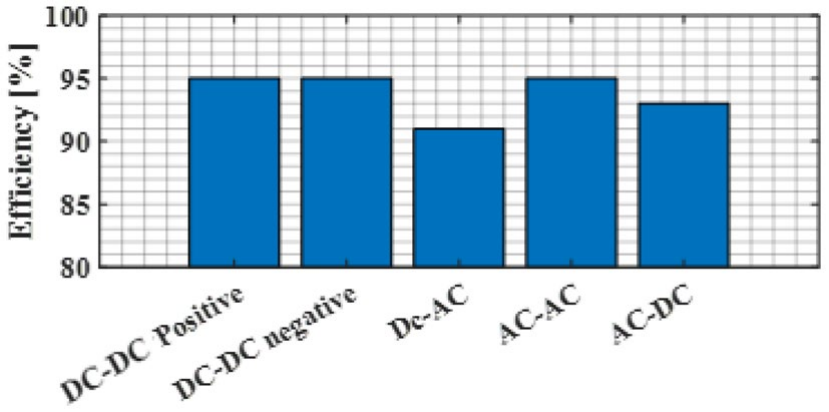
Efficiency of the proposed converter.

Figure 21 depicts the efficiency of each converter function with a duty cycle of 50% and a switching frequency of 5 kHz. For positive or negative DC–DC functions as well as for AC–AC functions, the efficiency is 95%, while for DC–AC and AC–DC, it is 91% and 93%, respectively.

## Conclusions

The paper introduced a power electronic circuit topology able to operate as a multi-function converter. The operation modes are described mathematically and modelled for small signal analysis using a state-space averaging technique. The frequency response analysis shows the effects of circuit parameters in terms bode-plot and pole-zero plane. The performance of the proposed circuit as a multi-function converter is evaluated theoretically by computer simulation and experimentally using a DSP-based laboratory model. The experimental results confirm those obtained by computer simulation and physically realize the successfully operation of the proposed circuit operating as a multi-function converter. The techniques and results introduced are of interest to power system engineers, forming a useful base and guide for designing reliable smart and micro power system grids.

## Supplementary Information

Supplementary Information.

## Data Availability

All data generated or analysed during this study are included in this published article.
