# Supplementary material for: Design, implementation and performance evaluation of multi-function boost converter
Source: Sci Rep. 2023 Mar 15;13:4276. doi: 10.1038/s41598-023-31293-5 (PMC10017816; doi:10.1038/s41598-023-31293-5)
Supplement: Supplementary file 1 — Supplementary Information. [file 41598_2023_31293_MOESM1_ESM.docx]

1. Appendix

11.1 Switching Losses Calculations

IGBT losses are mainly divided into two categories, the first one is the conduction losses which occurs during the normal on and off periods and depends on the saturation and cut-off values of the collector current and collector to emitter voltage. However, the other type of the losses is named as switching losses which depends primarily on the turn on and turn off characteristics of IGBT. The switching losses represents the dominant losses of the switch at switching operation as in many power electronics applications. The turn on and off times of IGBT can be expressed as follows:

$t_{on}=t_{d\left( on \right)}+t_{r}$ (A1)

$t_{off}=t_{d\left( off \right)}+t_{f}$ (A2)

Where $t_{on}$ is the turn on time of IGBT, $t_{d\left( on \right)}$ is the on delay time, $t_{r}$ is the rise time, $t_{off}$ is the turn off time of IGBT, $t_{d\left( off \right)}$ is the off delay time, and $t_{f}$ is the fall time. The time quantities expressed by equations (A1) and (A2) are depends on the switch manufacturing and can be obtained from IGBT datasheet.

To calculate the power losses of IGBT, the total switching period can be divided into 6-time intervals as shown in Fig. A1. These time intervals are on-delay time $t_{d\left( on \right)}$, rise time $t_{r}$, off-delay time $t_{d\left( off \right)}$, and fall time $t_{f}$, which are restricted to the on and off characteristics of the transistor and they are the times at which the switching losses occurs, normal conduction time $t_{n}$, and normal off time $t_{o}$ which are dependent on the duty ratio of the IGBT and the losses through these periods represents the conduction losses.

The conduction losses can be calculated from

$P_{l\left( con \right)}=\frac{1}{T}*(\int_{0}^{t_{n}} v_{ce\left( s \right)}*i_{c\left( s \right)} dt+\int_{0}^{t_{n}} v_{ce\left( cf \right)}*i_{c\left( cf \right)} dt)$ (A3)

Where $T$ is the switching period, $P_{l\left( con \right)}$ is the conduction losses, $v_{ce\left( s \right)}$ is the saturation value of the collector to emitter voltage, $i_{c\left( s \right)}$ is the saturation value of the collector current, $i_{c\left( cf \right)}$ is the cutoff value of the collector current, and $v_{ce\left( cf \right)}$ is the cutoff value of the collector to emitter voltage. However, the switching losses can be evaluated as:

$P_{l\left( sw \right)}=\frac{1}{T}*(\int_{0}^{t_{d\left( on \right)}} v_{ce\left( cf \right)}*i_{c\left( cf \right)} dt+\int_{0}^{t_{r}} v_{ce\left( r \right)}*i_{c\left( r \right)} dt+\int_{0}^{t_{d(off)}} v_{ce\left( s \right)}*i_{c\left( s \right)} dt+\int_{0}^{t_{f}} v_{ce\left( f \right)}*i_{c\left( f \right)} dt)$ (A4)

Where $P_{l\left( sw \right)}$ is the switching losses, $v_{ce\left( r \right)}$, and $i_{c\left( r \right)}$ are the collector to emitter voltage and collector current during the rising time respectively. $v_{ce\left( f \right)}$, and $i_{c\left( f \right)}$ are the collector to emitter voltage and collector current during the falling time respectively. For the sack of simplifying the calculations, let $v_{ce\left( r \right)}, i_{c\left( r \right)}, v_{ce\left( f \right)}, andi_{c\left( f \right)}$be stright lines as in [4]. Then equation (A4) can be written as:

$P_{l\left( sw \right)}=f_{sw}*(\frac{t_{f}\left( 2*i_{c\left( cf \right)}*v_{c\left( cf \right)}+i_{c\left( cf \right)}*v_{ce\left( s \right)}+i_{c\left( s \right)}*v_{ce\left( cf \right)}+2*i_{c\left( s \right)}*v_{ce\left( s \right)} \right)}{6}+\frac{t_{r}\left( 2*i_{c\left( cf \right)}*v_{c\left( cf \right)}+i_{c\left( cf \right)}*v_{ce\left( s \right)}+i_{c\left( s \right)}*v_{ce\left( cf \right)}+2*i_{c\left( s \right)}*v_{ce\left( s \right)} \right)}{6}+i_{c\left( cf \right)}*t_{d}*v_{ce\left( cf \right)}+i_{c\left( s \right)}*t_{s}*v_{ce\left( s \right)})$ (A5)

Where $f_{sw}$ is the switching frequency. If the conduction losses are neglected by putting $i_{c\left( cf \right)}$ and $v_{ce\left( s \right)}$to zeros. Then equation (A5) is simplified as:

$P_{l\left( sw \right)}=\frac{f_{sw}}{6}*(i_{c\left( s \right)*}v_{ce\left( cf \right)}\left( t_{r}+t_{f} \right))$ (A6)

Fig. A1. Turn on and turn off characteristics of IGBT. (a) Gate-Emitter voltage, (b) Collector-Emitter voltage, (c) Collector current, and (d) IGBT power loss.
